# Effect of Treatment of the Cholinergic Precursor Choline Alphoscerate in Mild Cognitive Dysfunction: A Randomized Controlled Trial

**DOI:** 10.3390/medicina60060925

**Published:** 2024-06-01

**Authors:** Anna Carotenuto, Vincenzo Andreone, Francesco Amenta, Enea Traini

**Affiliations:** 1 Telemedicine and Telepharmacy Centre, School of Medicinal and Health Products Sciences, University of Camerino, 62032 Camerino, Italy; annacarotenuto@gmail.com (A.C.); enea.traini@unicam.it (E.T.); 2 Neurology and Stroke Unit-Neurology, A. Cardarelli Hospital, 80131 Naples, Italy; vincenzo.andreone@aocardarelli.it

**Keywords:** cognitive dysfunction, mild cognitive impairment, choline alphoscerate, randomized controlled trial, study protocol

## Abstract

*Background and Objectives:* The focus on mild cognitive dysfunction in adults is of great interest, given the risk of worsening and conversion to dementia. Cognitive dysfunctions are characterized by a decrease in the weight and volume of the brain, due to cortical atrophy, with a widening of the grooves and flattening of the convolutions. Brain atrophy that mainly involves the hippocampus is related to the progression of cognitive impairment and the conversion from mild cognitive dysfunction to dementia. Currently, there is no treatment for MCI. Results from a trial on Alzheimer’s disease (ASCOMALVA trial) suggest that a sustained cholinergic challenge can slow the progression of brain atrophy typical of Alzheimer’s disease associated with vascular damage. This study intends to evaluate the efficacy of choline alphoscerate in patients with mild cognitive impairment (MCI) and associated vascular damage, in stabilizing and/or slowing brain atrophy typical of adult-onset cognitive dysfunction, and in improving and/or slowing the progression of cognitive and behavioral symptoms associated with MCI. *Materials and Methods*: This randomized controlled trial will recruit 60 patients that will be evaluated and randomized in a 1:1 ratio to receive choline alphoscerate (1200 mg/day) or placebo, for 12 months. Analyses will be carried out using SPSS vesion No 26 the Statistician in charge of this study, with the statistical significance level chosen as 0.05. *Discussion*: This trial may provide evidence about the efficacy of treatment with the cholinergic precursor choline alphoscerate in patients with mild cognitive dysfunction. The results of this study will be published in peer-reviewed journals. *Registration***:** EudraCT number: 2020-000576-38

## 1. Introduction

### Background

In recent years, attention to cognitive dysfunctions has increased. Given that Alzheimer’s disease (AD), and dementia in general, remains one of the most relevant problems for public health today, there is growing attention, on the part of both social and health systems, of tools and strategies to recognize the cognitive decline, and possibly distinguish “mild cognitive dysfunction” from “dementia” [1]. The focus on mild cognitive dysfunction in adults is of great interest, given the risk of worsening and conversion into overt dementia [2,3].

Mild cognitive impairment (MCI) is a clinical condition characterized by a difficulty in one or more cognitive domains, such as, memory, attention, or motivation, identified through neuropsychological tests, but not compromising the normal and daily activities of the patient [4].

There is currently no approved drug treatment for mild cognitive dysfunction defined by the current criteria that include evidence of objective cognitive impairment (typically memory), the preservation of general cognitive and functional abilities, and the absence of diagnosis of dementia [5]. Many of the drugs approved for the treatment of AD have been evaluated as potential therapies for the treatment of cognitive dysfunction without dementia. Systematic reviews and meta-analyses evaluating the efficacy of cholinesterase inhibitors (ChE-Is) for the treatment of cognitive impairment without dementia concluded that there is no convincing evidence that this class of compounds is effective on cognitive abilities [6,7,8]. A review that evaluated the effects of donepezil, rivastigmine, galantamine, or memantine on mild cognitive impairment concluded the ineffectiveness of all these drugs [6]. Vitamin E and donepezil did not show efficacy after three years of treatment [9], although donepezil may have reduced the likelihood of conversion in the first twelve months of treatment. Rivastigmine, galantamine, and many other drug categories did not show benefits [10,11,12]. Collectively, available evidence indicates that ChE-Is do not affect the conversion of MCI into dementia, or if effective, they display a very mild activity [13].

Furthermore, one of the main problems of ChE-I therapy is the decrease in the effectiveness of the treatment over time [14]. Another problem is that for particular categories of patients (very elderly subjects, that is, more than 85 years old, or patients with bradycardia, bronchial asthma, or chronic obstructive pulmonary disease), the use of ChE-I is not indicated. On the other hand, high doses of ChE-I are potentially associated with important side-effects [15].

Cholinergic precursors were among the first compounds used to treat AD [16].

The efficacy of increasing the level of acetylcholine in adult cognitive impairment is reported in various studies. The data supporting the drug’s activity are reported below in several review articles and meta-analyses [16,17].

Choline alphoscerate has no particular contraindications; the only one is hypersensitivity to the product. As a precursor of biological constituents, choline alphoscerate, even for prolonged administration, generally does not pose problems of tolerability. Studies on cognitive impairment, Alzheimer’s, dementia [18], Parkinson’s [19], or Cerebral Small Vessel Disease [20] showed that the incidence of side-effects in the group treated with choline alphoscerate is low and similar to those observed in the placebo group. The possible occurrence of nausea (probably attributable to a secondary dopaminergic activation) could require a reduction in the dosage and/or suspension of the drug.

Choline alphoscerate is, among the clinically tested cholinergic precursors, the compound that has shown the greatest efficacy, as well as good tolerability in patients with mild- and moderate-phase AD and with associated vascular damage [16]. Since choline alphoscerate crosses the blood–brain barrier easily, it likely acts as an active choline donor in the brain and has been shown to exert neuroprotective effects in experimental animals with vascular brain injury. Based on these considerations, a study called ASCOMALVA (association between the cholinesterase inhibitor donepezil and the cholinergic precursor choline alphoscerate in Alzheimer’s disease with cerebrovascular injury) recruited patients with AD associated with vascular damage, a patient population characterized by marked cholinergic hypofunction [21,22]. This study has shown that the group of patients treated with donepezil alone (control group) was more impaired on cognitive tests: the Mini-Mental State Examination (MMSE) and Alzheimer’s Disease Assessment Scale—Cognitive Subscale.

(ADAS-Cog) scores, functional scores, and the Instrumental Activities of Daily Living (IADL) were compared to the group treated with the combination choline alphoscerate and donepezil (experimental group) [18]. Moreover, the ASCOMALVA study has shown a greater benefit of the association of donepezil plus choline alphoscerate versus donepezil alone on behavioral disorders [23], particularly on apathy [24], with a significant reduction in the stress of the caregiver. Within the ASCOMALVA study, the difference in the brain volume of the two treatment groups was evaluated with the analysis of 3-year Magnetic Resonance Imaging (MRI) using voxel morphometry techniques. Results of this study have shown that the rate of atrophy progression was significantly higher in the group that received only donepezil than in the group that received the combination choline alphoscerate and donepezil (experimental group). The reduction in atrophy in the hippocampal and amygdaloid areas is consistent with the clinical efficacy demonstrated in terms of the reduction in cognitive loss (MMSE and ADAS-Cog scores) [18]. These results suggest that the marked cholinergic challenge induced by treatment with cholinergic precursors ChE-I + choline alphoscerate can slow the progression of brain atrophy typical of AD associated with vascular damage. Based on these results, this study aims to evaluate the effectiveness of the cholinergic precursor, choline alphoscerate, in subjects with mild cognitive dysfunctions with associated vascular damage.

## 2. Methods

### 2.1. Objectives of Study

#### 2.1.1. Primary Objective

This study intends to evaluate the slowing and/or stabilization of atrophy of the hippocampus, entorhinal cortex, neocortex, and ventricular dilation induced by treatment with the cholinergic precursor choline alphoscerate in patients with mild cognitive dysfunction with associated vascular damage.

#### 2.1.2. Secondary Objectives

This study intends to evaluate whether the effectiveness of the cholinergic precursor choline alphoscerate is superior to that of placebo in the stability and/or improvement in cognitive abilities.

Functional performances and changes in mood and motivation will also be monitored. The safety and tolerability of the study drug will be evaluated.

#### 2.1.3. Primary Endpoint

The volume (mm^3^) of the hippocampus, entorhinal cortex and neocortex and the dilation of lateral ventricles determined from T1-weighted MRI will be analyzed at 12-month intervals. Treatment will be deemed successful if atrophy of the areas is significantly lower than that of the control group at 12 months. Cognitive impairment will be measured using the MMSE scale at the final visit compared to baseline, and treatment will be successful if MMSE is significantly higher than that of the control group at 12 months

#### 2.1.4. Secondary Endpoints

Cognitive abilities will be assessed through the use of neuropsychological scales that evaluate the cognitive performance of patients (executive, memory, visual-constructive, linguistic, and attentional functions) at 6 months and at end of this study compared to the baseline visit. Treatment will be successful if cognitive performances are significantly higher than those of the control group at 12 months. Functional performances and changes in mood and motivation will be evaluated with specific neuropsychological tests, and treatment will be successful if functional performances are significantly higher and behavioral impairment significantly lower than those of the control group at 12 months.

#### 2.1.5. Safety Endpoint

The safety and tolerability of the study drug will be evaluated through the detection of adverse events (AEs), laboratory parameters, vital signs, and physical examination throughout the course of this study.

### 2.2. Study Population

A total of 60 patients, with mild cognitive dysfunction, meeting the following selection criteria will receive the study drug/placebo.

#### 2.2.1. Inclusion Criteria

These include the patient being able to understand and sign the informed consent or the informed consent being signed by a family member/caregiver.

- Age ≥ 65 years.
- Memory disorder presence evaluated by neuropsychological testing.
- MMSE: score ≥ 24.
- Clinical Dementia Rating (CDR) = 0.5.
- Sufficient education to enable the patient to read, write, and communicate effectively.
- Independent patient in daily, family, work, and/or social activities.
- Cooperative patient and able to complete all aspects of this study alone or with the help of a family member.
- Patient living with or in contact with a family member/caregiver who cooperates in the efficacy evaluation.
- MRI performed within 6 (six) months prior to enrolment.
- Presence of at least 2 (two) vascular risk factors including systemic arterial hypertension; diabetes mellitus; obesity; heart disease (e.g., atrial fibrillation); dyslipidemia; hyperhomocysteinemia; tobacco addiction; previous cerebrovascular events; and family history of cardio-cerebrovascular diseases.

#### 2.2.2. Exclusion Criteria

- MMSE: score < 23.
- Clinical Dementia Rating (CDR) > 0.5.
- All decompensated cardiac disorders.
- Chronic renal failure.
- Severe hepatic insufficiency
- Incorrect dysthyroidism (level T3 different from 1.1–2.6 nmol/L, T4 different from 60–150 nmol/L).
- Serious ongoing developmental systemic pathologies (e.g., malignancies).
- Any advanced, progressive, or unstable disease that, in the opinion of the investigator, could interfere with efficacy or safety assessments or that could put the patient at risk by participation in the study.
- Psychiatric disorders or intellectual disability (psychosis, dissociative syndrome).
- Alcohol/drug/substance abuse or dependence.
- Diagnosis of major depression according to DSM V tr (Diagnostic and Statistical Manual of Mental Disorders Text Revision), except in cases successfully treated with a stable dose of an antidepressant (non-anticholinergic) for at least 4 weeks prior to recruitment.
- Any contraindication to treatment or intolerance to choline alphoscerate.
- Patient involved in other clinical trials.

#### 2.2.3. Withdrawal Criteria

Patients must be withdrawn under the following circumstances:

- Patient’s wish to be withdrawn from the study (withdrawal of consent).
- Patient is lost to follow-up.
- Major protocol violation considered relevant by the investigator.
- The protocol therapy may be discontinued if the patient is judged to be intolerant to the study drug.
- The patient is not able to complete the 12 months’ treatment (<80% compliance).

If, during this study, a full-blown clinical picture of dementia emerges, the patient will be treated with the therapies indicated for this pathology and prematurely discontinued from the study (drop-out) due to disease progression. The data of patients enrolled but not evaluable will, in any case, be collected and entered into the study database. Any discontinuation must be recorded in the case report form by the investigator, who will indicate the date and reason(s) for withdrawal.

## 3. Trial Design

This is a non-profit, monocentric, prospective, randomized in double-blind, controlled vs. placebo, phase IV study. Taking into account that no treatments have been so far authorized with the specific indication for the MCI, the majority of symptoms typical of MCI are those for which choline alphoscerate has obtained the marketing authorization. In view of this, we are identifying this study as a phase IV investigation. A total of 60 patients with mild cognitive dysfunction and associated vascular damage will be evaluated and randomized in a 1:1 ratio of choline alphoscerate (1200 mg/day) or placebo, for 12 months. The study duration is estimated to be 24 months for each enrolled patient: 12 months for the enrolment period, max. 21 days for the screening period, 12 months for the treatment period, and, finally, the project completion of data collection: LVLP (Last Visit Last Patient).

Patients will be informed about the objectives, procedures, and possible risks and will be asked to sign and to date the informed consent for inclusion in this study. They will be visited and assessed for inclusion and exclusion criteria. During screening, patients will undergo the following assessments:

- Demographic data (age, sex).
- Physical examination, vital signs, and blood pressure.
- Medical history.
- Intake of previous/concomitant therapies.
- Laboratory tests: complete blood count with formula; AST; ALT; creatinine; glycemia; azotemia; total bilirubin; total and HDL cholesterol; triglycerides; thyroid hormones FT3, FT4, and TSH; vitamin B12; and folic acid (sampling performed within 1 month of screening will be accepted).
- Electrocardiogram (ECG) (examination performed within 3 months of screening will be accepted).
- MRI (examination performed within 6 months of screening will be accepted).

Cognitive functions will be evaluated through the administration of the following neuropsychological tests: Mini-Mental State Examination (MMSE); Montreal Cognitive Assessment (MOCA); Memory of the Figure of Rey; Test of Corsi; Verbal Span; Immediate and Deferred Re-enactment of Rey’s 15 words; Free and Cued Selective Reminding Test (FCRST); Clock Drawing Test; Copy of Rey’s Figure; Frontal Assessment Battery (FAB); Phonemic verbal fluency test; Semantic verbal fluency test; Attentive Matrices Test; Beck Depression Inventory (BDI); Apathy Evaluation Scale (AES); Basic Activities of Daily Living (BADL); Instrumental Activities of Daily Living (IADL); and study drug dispensation. The BADL and IADL scales will be administered to the patient’s family member/caregiver.

Baseline Visit (Visit V0) is scheduled within 3 weeks from screening. This visit might overlap with screening if all data requested for enrolment are available. During this visit, the investigator will check for compliance with the inclusion/exclusion criteria. The patients who meet the criteria will be randomized to receive the study treatment (choline alphoscerate or placebo). At the end of the visit, patients meeting the inclusion criteria will be provided with a sealed and coded small box containing the study treatment.

Visit 1 is scheduled at 3 months from baseline visit (±3 days). At this visit, for a compliance check, all used and unused containers of treatment should be returned. The patients will therefore receive choline alphoscerate/placebo for the next 3 months of treatment.

**Figure 1:**
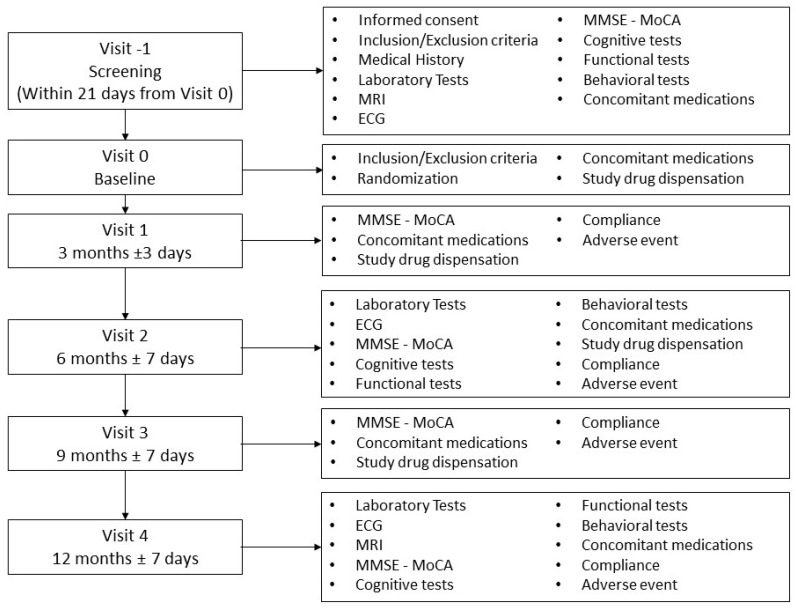
Flowchart. MRI: Magnetic Resonance Imaging; ECG: Electrocardiogram; MMSE: Mini Mental State Evaluation; MoCA: Montreal Cognitive Assessment. Cognitive tests: Verbal Span; Immediate and Deferred Rey’s 15 words; Test of Corsi; Free and Cued Selective Reminding Test; Phonemic verbal fluency test; Semantic verbal fluency test; Attentive Matrices Test; Rey Figure; Clock Drawing Test; Frontal Assessment Battery; Functional test: Basic Activities of Daily Living; Instrumental Activities of Daily Living; Behavioral test: Apathy Evaluation Scale; Beck Depression Inventory.

Follow-up will be carried out at 3, 6, 9, and 12 months. The evaluation will be according to the flowchart reported in Figure 1. The patient will perform MRI at enrolment and at 12 months for a detailed measurement of the levels of atrophy in the areas of interest.

### Randomization

Patients will be randomly assigned to ARM A (placebo) or ARM B (choline alphoscerate) with a 1:1 ratio. The allocation to the assigned treatment ARM will remain unknown to the patients, to the Principal Investigator and/or her delegates, and to the personnel in charge of the data management and the statistical analyses until the database lock. The randomization list will be prepared by an Independent Statistician who will collaborate with the unblended responsible for the preparation of the experimental drug for the randomization list management, using a computer random number generator. The randomization will be performed at Visit 0 (baseline), after the inclusion/exclusion criteria check.

## 4. Statistical Analysis

### 4.1. Sample Size

In accordance with the primary study objective, the estimation of sample size is based on the variation in the volume of the hippocampus. There is no study evaluating brain atrophy in MCI patients treated with cholinergic drugs. The clinical hypothesis to be verified is a reduction in hippocampal volume. This value has shown the ability to discriminate patients between AD, MCI, and the healthy control [25].

Based on this study, the mean difference in these groups was 0.7 cm^3^ with a maximum standard deviation of 0.6 [25]. Fixing α = 0.05, β = 0.05 (i.e., power = 95%), and SD = 0.6 (chosen as the higher value observed in the reference study) and conducting a two-sided t test, 42 patients (i.e., 21 patients in each arm) are sufficient for detecting a difference of 2 points in MMSE. To reduce the influence of drop-out patients on the estimate of the treatment effect size, a further 25% patients will be added. Based on these calculations, a total of 60 patients (30 in each arm) will be randomized.

### 4.2. Methods

All the variables will be descriptively analyzed by treatment and visit (mean, median, standard deviation, interquartile range for continuous variables, frequency distribution for categorical variables). Efficacy analysis will be applied in all populations. Results from the ITT (Intention To Treat) population will be considered the primary ones.

Analyses will be performed using SPSS by the Statistician in charge of this study (different from the above-mentioned Independent Statistician). The statistical significance level chosen is equal to 0.05.

### 4.3. Analysis

The treatment comparison on the difference between the two arms in the changes in brain hippocampal volume at the final visit from baseline will be performed using Repeated-Measures ANOVA.

The test will be conducted at the standard 0.05 significance level. In case of a non-normal distribution, the data will be normalized through a log-transformation. A non-parametric method will be used in case the normality will not be observed after the data transformation.

The treatment effect will be estimated in terms of the difference in changes at the final visit from the baseline between the two treatments, and the corresponding two-sided 95% Confidence Interval (CI) will be provided. All the secondary endpoints will be analyzed according to their standard methods. All the statistical methods will be detailed on the Statistical Analysis Plan which will be finalized before the database freezing. Statistical analysis of the safety data will address the safety population. No method for replacing missing values will be applied, and all the missing datapoints will be removed from analysis according to pairwise exclusion. The MedDRA dictionary will be used for coding AEs. AEs will be summarized by the System Organ Class (SOC) and Primary Preferred Term (PPT). They will also be stratified by seriousness and relationship with the study treatment. All abnormalities noted on the physical examination at enrolment and during this study will be descriptively analyzed as the frequency distribution. Previous and concomitant medications will be coded using the WHO dictionary with the ATC codes. Concomitant diseases at study entry will be coded with the MedDRA dictionary. These data will be descriptively analyzed as frequency distributions.

### 4.4. Setting and Data Management

Recruitment of participants will take place at the Memory Unit of the Department of Neurology at the Cardarelli Hospital, Naples. Data collection will be carried out on-site. Supervision, organization, and informatics support and statistics will be provided by the Clinical Research Centre of Camerino University, Camerino, Italy. Clinical data will be input into a computerized file on the net developed for protocols. Each researcher can access the clinical files using a personal password. The identity of subjects was not kept in the WEB but was taken while protected by the investigating center only. The numerical list was sent to the Clinical Research Centre of Camerino University for randomization purposes and for allotting patients to one of the two treatments planned (placebo or choline alphoscerate). The data will be entered by the investigator or his delegate in a special database. The electronic system, which will be created and validated by the Statistician, will include the data entry pages prepared based on the study flow diagram. Medical information of the subject taking part in the trial will be treated in accordance with the General Data Protection Regulation (GDPR). Collected data will be digitally stored in a seamless plug-and-play technology archive with password protection and hardware encryption to strengthen content security. The portable unit will be stored in a locked place. Data cleansing will be performed by the Data Manager of this study. Before freezing the data, the biologist in charge of this study will code the drugs according to the following dictionary: WHO-ATC for drugs. At the end of this study, the Data Manager will take care of freezing the database. The Data Manager will provide the Biostatistics Unit with the clean database for statistical analysis.

## 5. Ethical Considerations

The protocol and its annexes were approved by the competent Independent Ethics Committee “Federico II University—Cardarelli Hospital” and by the Italian Medicines Agency (AIFA). This study will be conducted according to the ICH Guideline for Good Clinical Practice, as applicable. This study will also be conducted in accordance with the Ministerial Decree of 17 December 2004 (non-profit study). All patients will be informed of the aims of this study, the procedures, and possible hazards to which they will be exposed, and the mechanism of treatment allocation. They will be informed as to the strict confidentiality of their patient data, but that their medical records may be reviewed for study purposes by authorized individuals other than their treating physician. It will be emphasized that participation is voluntary and that the patient is allowed to refuse further participation in the protocol whenever they want. This will not prejudice the patient’s subsequent care. Documented informed consent must be obtained for all patients included in this study before they are registered at the Center. This must be performed in accordance with the national and local regulatory requirements. The trial will be concluded with the execution of the LVLP. Any changes from the approved protocol will be submitted to the Ethics Committee for their review and approval.

The protocol was redacted according to SPIRIT reporting guidelines [26].

## 6. Discussion

The CARL study (choline alphoscerate in mild cognitive dysfunction) intends to evaluate the efficacy of choline alphoscerate in 60 patients with mild cognitive impairment (MCI) and associated vascular damage, such as the ability to induce stability and/or slow atrophy of the hippocampus, entorhinal cortex, and neocortex and lateral ventricle dilation.

The study of the measurement of the human brain through magnetic resonance is very important for the diagnosis of cognitive impairment [27]. Structural MRI studies have frequently focused on hippocampal volume [28,29,30] and global brain atrophy [30,31,32]. Brain atrophy mainly involving the hippocampus correlates the progression of cognitive impairment, with the conversion from mild cognitive impairment to overt AD [33]. In particular, the first region to degenerate in AD, even before the onset of symptoms, is the hippocampus, followed by other brain areas in later stages. The evaluation of hippocampal volume provides a potential indicator for discriminating patients between AD, MCI, and healthy controls [25]. In fact, the volume of the hippocampus is one of the main biomarkers of AD and the morphometric analysis of this parameter based on magnetic resonance is one of the most used systems to evaluate the progression of AD [34]. The various shades of gray of the hippocampus and surrounding areas allow the degree of atrophy to be estimated. In general, hippocampal atrophy was assessed by manual MRI image analysis, both with manual analysis and with an automated system [35]. It is preferable to use high-performance automated systems for MRI image analysis to avoid mistakes caused by subjective evaluation by investigators. The MRI exam allows the detailed visualization of the hippocampus and, after acquiring a high-resolution 3D MRI sequence of the human brain, the images can be processed using specific algorithms and then analyzed according to validated protocols [34,36,37,38]. The previous study on AD patients with associated vascular damage (ASCOMALVA) evaluated the difference in brain volume through the MRI study on 56 patients. The results showed that the group treated with donepezil and choline alphoscerate showed less pronounced gray matter atrophy than the control group treated with donepezil alone, in the frontal and temporal lobes, in the hippocampus, and in the amygdala. The reduction in atrophy in the hippocampal and amygdaloid area is consistent with the clinical efficacy demonstrated in terms of the reduction in cognitive loss (MMSE and ADAS-Cog score) and in the improvement in behavioral parameters, as well as with the improvement in the perception of the caregiver (NPI). The less pronounced atrophy of the hippocampus and amygdala occurring in patients receiving choline alphoscerate in addition to standard treatment with donepezil suggests that a marked cholinergic problem induced by treatment of the cholinergic precursor ChE-I may slow the progression of brain atrophy, typical of AD [18]. The CARL study will extend the results of ASCOMALVA to subjects with mild cognitive impairment with associated vascular damage.

## Data Availability

Patient health data cannot be made available, for privacy reasons. However, the statistical codes and all study materials will be available from the corresponding author on reasonable request, and any questions about the procedure are welcome. Contact for more information on the study: francesco.amenta@unicam.it. All authors have approved the manuscript for submission. The content of the manuscript has not been published or submitted for publication elsewhere.

## Abbreviations

AD	Alzheimer’s Disease
ADAS-Cog Alzheimer’s	Disease Assessment Scale—Cognitive Subscale
AEs	Adverse Events
AES	Apathy Evaluation Scale
AIFA	Agenzia Italiana del Farmaco
ASCOMALVA	Association between the cholinesterase inhibitor donepezil and the cholinergic precursor choline alphoscerate in Alzheimer’s disease with cerebrovascular injury
BADL	Basic Activities of Daily Living
BDI	Beck Depression Inventory
ChE-I	Cholinesterase Inhibitor
CDR	Clinical Dementia Rating
CRF	Case Report Form
DSM V tr	Diagnostic and Statistical Manual of Mental Disorders Text Revision
ECG	Electrocardiogram
FAB	Frontal Assessment Battery
FCRST	Free and Cued Selective Reminding Test
IADL	Instrumental Activities of Daily Living
ITT	(Intention To Treat)
LVLP	Last Visit Last Patient
MCI	Mild Cognitive Impairment
Microsoft	Microsoft Excel
MOCA	Montreal Cognitive Assessment
MMSE	Mini-Mental State Evaluation
MRI	Magnetic Resonance Imaging
NPI	Neuropsychiatric Inventory
PPT	Primary Preferred Term
Protocol Final v1.0 date	15 November 2021 12 of 83
SOC	System Organ Class
SPSS IBM^®^	SPSS^®^ software
